# Application of machine learning for mass spectrometry-based multi-omics in thyroid diseases

**DOI:** 10.3389/fmolb.2024.1483326

**Published:** 2024-12-17

**Authors:** Yanan Che, Meng Zhao, Yan Gao, Zhibin Zhang, Xiangyang Zhang

**Affiliations:** ^1^ School of Pharmaceutical Science and Technology, Tianjin University, Tianjin, China; ^2^ Department of General Surgery, Tianjin First Central Hospital, Tianjin, China

**Keywords:** mass spectrometry, proteomics, metabolomics, multi-omics, thyroid diseases, machine learning

## Abstract

Thyroid diseases, including functional and neoplastic diseases, bring a huge burden to people’s health. Therefore, a timely and accurate diagnosis is necessary. Mass spectrometry (MS) based multi-omics has become an effective strategy to reveal the complex biological mechanisms of thyroid diseases. The exponential growth of biomedical data has promoted the applications of machine learning (ML) techniques to address new challenges in biology and clinical research. In this review, we presented the detailed review of applications of ML for MS-based multi-omics in thyroid disease. It is primarily divided into two sections. In the first section, MS-based multi-omics, primarily proteomics and metabolomics, and their applications in clinical diseases are briefly discussed. In the second section, several commonly used unsupervised learning and supervised algorithms, such as principal component analysis, hierarchical clustering, random forest, and support vector machines are addressed, and the integration of ML techniques with MS-based multi-omics data and its application in thyroid disease diagnosis is explored.

## 1 Introduction

The thyroid gland is a small, butterfly-shaped gland located at the base of the neck (Sofia et al., 2019; Mullur et al., 2014). It plays a crucial role in regulating various metabolic processes by secreting hormones (Sofia et al., 2019; Mullur et al., 2014). Thyroid disease refers to various diseases affecting the thyroid gland, categorized into functional and neoplastic diseases (Vanderpump, 2011; Zhang et al., 2022). Functional diseases are classified as hyperthyroidism or hypothyroidism, whereas neoplastic diseases are classified as benign or malignant (Zhang et al., 2022).

In the field of neoplastic diseases, tumors are classified as benign tumors, low-risk neoplasms, and malignant neoplasms according to prognostic risk categories (Basolo et al., 2023). Thyroid cancer refers to malignant tumors, originating from follicular or parafollicular thyroid cells, which can metastasize to other places in the body (Omur and Baran, 2014). Thyroid cancer is one of the most common endocrine neoplasia, and its incidence has been on the rise in the past 40 years, disproportionately affecting women (Chen et al., 2023a; Guarino et al., 2010).

According to “The 5th edition of the World Health Organization (WHO) classification of endocrine tumors” which was released in 2022, thyroid cancer exists in several forms (Schneider and Chen, 2013), including differentiated thyroid cancer (DTC), undifferentiated thyroid cancer, and medullary thyroid cancer (MTC). DTC, the most prevalent type of thyroid malignancy, primarily includes papillary thyroid carcinoma (PTC), follicular thyroid carcinoma (FTC), and oncocytic thyroid carcinoma, with PTC accounting for 85% –90% of all DTC cases (Omur and Baran, 2014; Caria et al., 2019). Thyroid cancer presents a complex and clinically significant challenge. To explore the molecular mechanisms of thyroid cancer, researchers have increasingly turned to omics approaches.

Omics is a technique for the comprehensive evaluation of different classes of biomolecules, including genomics, transcriptomics, proteomics, metabolomics, and others (Babu and Snyder, 2023). Using only one type of data to understand the characteristics and complications of a disease is not enough. Recently, exhaustive exploration through multi-omics strategies has garnered increasing attention among analytical chemists (Kappler and Lehmann, 2019). Advances in various omics technologies, such as proteomics and metabolomics, coupled with enhanced computing capabilities, have paved the way for innovative integration of diverse omics data (Babu and Snyder, 2023). With the rapid development of high throughput sequencing and multi-omics, biomedical research has increasingly adopted a combination of multi-omics technologies. Multi-omics strategies aim to scrutinize the same samples using two or more omics methods, integrating diverse omics data to reveal coherent associations and attain a comprehensive, holistic understanding of biomedical processes (Kappler and Lehmann, 2019).

Mass spectrometry (MS) is crucial for studying multi-omics. It is a high-throughput analytical technology that can quantify countless molecules, from metabolites and lipids to peptides and proteins (Zhao et al., 2022a; Leung Kwan et al., 2021). This analytical technology aids in discovering biomarkers, understanding diseases at the molecular level, and provides a new perspective in the biological field (Leung Kwan et al., 2021). As an emerging approach of biomarker discovery, MS-based multi-omics plays a significant role in the early diagnosis and screening, classification, and prognosis of diseases. However, the large amounts of data generated by high-throughput technologies require specialized data analysis strategies (Zhao et al., 2022b).

Machine learning (ML) is a driving force behind data integration in systems biology (Alber et al., 2019). Through data-driven bioinformatics analysis of MS-based multi-omics data, ML serves as a powerful tool for revealing the intrinsic mechanisms of various biological events (Leung Kwan et al., 2021). The combination of MS-based multi-omics and advanced data integration approach holds promise for deeper investigation of complex biological processes.

In this review, we presented the detailed review of applications of ML for MS-based multi-omics in thyroid disease. In literature previously published, applications of ML in thyroid disease or applications of MS-based multi-omics in thyroid disease are reviewed, but no one has combined them into a comprehensive review. This review can provide new insights to the people who focuses on applications of combining ML with MS-based multi-omics in thyroid disease. It is primarily divided into two sections. The first section briefly introduces MS-based multi-omics, mainly proteomics and metabolomics, and their applications in clinical diseases. The second section addresses a comprehensive overview of ML models, and explores the integration of ML techniques into MS-based multi-omics data, and its application in thyroid disease diagnosis.

## 2 Mass spectrometry-based multi-omics in thyroid diseases

**FIGURE 1:**
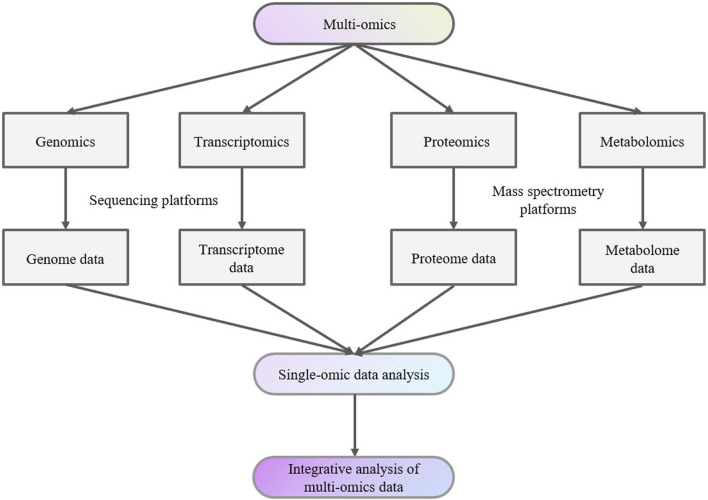
Multi-omics analysis flow chart.

Data from various studies, including genomics, transcriptomics, proteomics, and metabolomics studies together are denoted as “multi-omics” data (Figure 1). Individual datasets from these “-omics” studies can serve as valuable biomarkers for studying, exploring, and understanding the traits and complexities of biological organisms (Manochkumar et al., 2023).

MS plays a crucial role in multi-omics research by detecting metabolites or proteins in samples (Qiu et al., 2023). The use of mass spectrometry technology for detecting metabolites or proteins in samples can identify thousands of proteins or metabolites across a substantial volume of samples (Kowalczyk et al., 2020). This high-throughput approach not only improves our ability to identify molecular signatures but also helps us gain a more comprehensive understanding of the intricate biological processes within organisms (Leung Kwan et al., 2021).

### 2.1 Mass spectrometry-based proteomics

Oncogenesis is associated with changes in the levels of various proteins involved in cell proliferation, migration, and apoptosis (Migisha et al., 2020). Proteomics enables the maximum identification and quantification of all proteins in cells or tissues, establishes the connection between genes and their corresponding protein products, and provides information about proteins, including their subcellular localization, post-translational modifications, and interactions with other proteins, aiming to reveal the mechanisms behind their biological functions (Manochkumar et al., 2023; Chen et al., 2023b; Kang et al., 2022). The analysis of the proteome can provide valuable insights into the fundamental molecular mechanisms of diseases, responses to therapy, and the identification of diagnostic, predictive biomarkers and prognostic crucial for precision medicine (Ball et al., 2023).

**FIGURE 2:**
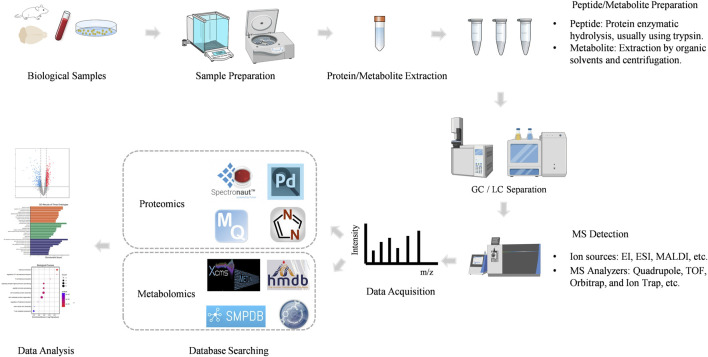
The workflow of MS-based proteomics and metabolomics analysis.

Mass spectrometry (MS) is an analytical technique that measures the mass-to-charge ratio (m/z) of ionized molecules. The basic components of a mass spectrometer include the ion source, mass analyzer, and detector. Proteins or peptides are ionized in the ion source, separated based on their m/z in the mass analyzer, and detected to generate a mass spectrum. This mass spectrum provides detailed information about the molecular weight and structural characteristics of the analyte. Mass spectrometry-based proteomics mainly includes five processes (Figure 2).

#### 2.1.1 Sample preparation

The proteomics workflow begins with the preparation of biological samples. Proteins are extracted from the sample, often followed by enrichment or fractionation to reduce complexity. This step is critical for ensuring the accurate identification and quantification of proteins.

The proteins are then digested into smaller peptides, typically using an enzyme like trypsin. This peptide mixture is more amenable to analysis by MS.

#### 2.1.2 Peptide ionization

Peptides are ionized in the ion source, which can be achieved through various techniques. The most common ionization methods in proteomics are Electrospray Ionization (ESI) and Matrix-Assisted Laser Desorption/Ionization (MALDI).

ESI is widely used for liquid chromatography-mass spectrometry (LC-MS) and generates ions by applying a high voltage to a liquid sample, producing charged droplets that release ions. MALDI involves embedding the sample in a matrix that absorbs laser energy, leading to the ionization of peptides.

#### 2.1.3 Mass analysis

The ionized peptides are introduced into the mass analyzer, where they are separated based on their m/z ratio. There are several types of mass analyzers, including Quadrupole, Time-of-Flight (TOF), Orbitrap, and Ion Trap, each offering different advantages in terms of resolution, accuracy, and speed.

High-resolution mass analyzers, such as the Orbitrap and TOF, are particularly valuable in proteomics for their ability to distinguish between ions with very similar m/z ratios, enabling the identification of complex peptide mixtures.

#### 2.1.4 Peptide fragmentation

To obtain sequence information, peptides are often subjected to fragmentation in the mass spectrometer. Tandem mass spectrometry (MS/MS) involves two stages of mass analysis: the first stage selects a precursor ion, which is then fragmented, and the second stage analyzes the resulting fragment ions.

The fragmentation patterns are characteristic of the peptide’s amino acid sequence, allowing for the identification of the peptide and inference of the protein from which it originated (Searle et al., 2020).

#### 2.1.5 Data analysis

The mass spectrometry data are processed using sophisticated bioinformatics tools. Software such as MaxQuant, Proteome Discoverer, DIA-NN, Peaks, Spectronaut and Mascot matches the obtained mass spectra to theoretical spectra derived from protein databases, enabling protein identification.

Quantitative proteomics can be achieved through various techniques, including label-free quantification, stable isotope labeling (e.g., SILAC, iTRAQ), and tandem mass tags (TMT). These approaches allow for the relative or absolute quantification of proteins across different samples.

The development of highly sensitive and high-throughput MS platforms over the past decade means that it is now possible to identify and quantify thousands of proteins from large numbers of biological samples. Rapid advancements in MS and data analysis strategies have significantly enhanced proteomics research worldwide (Halder et al., 2021). MS-based proteomics is increasingly recognized as a widely adopted technique for characterizing proteomes (Migisha et al., 2020). Proteomics research can be divided into untargeted proteomics and targeted proteomics. Untargeted proteomics is also called discovery proteomics, which detects differential proteins in different samples by detecting proteins as many as possible. The research objects of untargeted proteomics are uncertain, and are often all the protein or peptide components contained in the sample, which are relatively large in number. Targeted proteomics is the quantitative detection of target proteins. The research objects of targeted proteomics are specific and the number is relatively small. Compared with untargeted proteomics, it has greater sensitivity and accuracy and is often used for verification analysis of biomarkers. Data-dependent acquisition (DDA) and data-independent acquisition (DIA) are the two primary MS strategies for untargeted proteomics (Qian et al., 2023). DDA is a traditional MS-based proteomics analysis method. In DDA, in the second stage of tandem mass spectrometry, a small number of peptides are selected for fragmentation within a narrow range of mass-to-charge ratio (m/z) signal intensity (Hu et al., 2016). DIA is another MS-based proteomics analysis method. DIA divides the entire full scan range of the mass spectrometer into several windows and then fragments all peptide precursors within each window simultaneously to generate a comprehensive MS^2^ spectrum (Kawashima et al., 2019; Wang et al., 2022a).

### 2.2 Mass spectrometry-based metabolomics

Metabolomics is to detect and qualitatively and quantitatively analyze the dynamic changes of metabolites of organisms, tissues or cells before and after a specific stimulus or interference which was initially introduced in 1999 by Jeremy-Nicholson and is an emerging research field (Nicholson et al., 1999; Wang et al., 2023a). The research objects are metabolites, which are mostly small molecule substances with a molecular mass range of ≤1,000 Da, such as small organics: acids, amino acids, nucleotides, sugars, lipids, vitamins, etc. Metabolites are the end products of cellular processes and can directly reflect the physiological state of an organism. Liquid chromatography coupled to mass spectrometry (LC-MS) was first used to study thyroid cancer in serum samples in 2011 (Yao et al., 2011). DIA workflow was applied for metabolomics in 2017 (Zhou et al., 2017).

MS-based metabolomics involves the separation, detection, and characterization of metabolites, providing comprehensive coverage of the metabolome. The workflow is shown in Figure 2.

#### 2.2.1 Sample preparation

The first step in MS-based metabolomics involves the preparation of biological samples. Metabolites can be extracted from various biological matrices, such as plasma, urine, tissues, or cell cultures, using extraction methods optimized for different classes of metabolites.

Sample preparation is critical to preserving the integrity of the metabolome and avoiding contamination or degradation. The extracted metabolites are often subjected to concentrate to improve the detection of low-abundance compounds.

#### 2.2.2 Metabolite separation

Prior to mass spectrometric analysis, metabolites are typically separated using chromatographic techniques to reduce sample complexity. The most common techniques are Gas Chromatography (GC) and Liquid Chromatography (LC).

Gas Chromatography-Mass Spectrometry (GC-MS) is particularly well-suited for analyzing volatile and semi-volatile compounds. In GC-MS, metabolites are vaporized and separated in a gas phase before being ionized and detected by a mass spectrometer.

Liquid Chromatography-Mass Spectrometry (LC-MS) is more versatile and can handle a broader range of metabolites, including polar, non-volatile, and thermally labile compounds. LC-MS separates metabolites in a liquid phase based on their interaction with the stationary phase and then ionizes them for mass spectrometric detection.

#### 2.2.3 Ionization of metabolites

The ionization of metabolites is a crucial step in mass spectrometry, as it converts neutral molecules into charged ions that can be detected. Common ionization methods include Electrospray Ionization (ESI) and Atmospheric Pressure Chemical Ionization (APCI) for LC-MS, and Electron Ionization (EI) for GC-MS.

ESI is widely used in LC-MS due to its ability to ionize a wide range of metabolites, particularly those that are polar and easily ionizable. ESI produces ions by applying a high voltage to the liquid sample, resulting in charged droplets that release ions as they evaporate.

EI, commonly used in GC-MS, involves bombarding gas-phase molecules with high-energy electrons, leading to ionization and fragmentation. The resulting fragment ions provide structural information about the metabolite.

#### 2.2.4 Mass analysis and detection

Once ionized, metabolites are introduced into the mass analyzer, where they are separated based on their m/z ratio. Unlike proteomics, metabolomics is divided into positive and negative ion modes due to the different properties of the compounds. Various mass analyzers are used in metabolomics, including Quadrupole, Time-of-Flight (TOF), Orbitrap, and Ion Trap analyzers.

High-resolution mass analyzers, such as the Orbitrap and TOF, are particularly valuable in metabolomics for their ability to accurately measure the m/z of metabolites and distinguish between compounds with very similar masses.

#### 2.2.5 Data acquisition and processing

The mass spectrometer generates a mass spectrum, which provides information on the m/z ratios and intensities of detected ions. This data is then processed using specialized software to identify and quantify metabolites.

The identification of metabolites is typically performed by matching the acquired mass spectra against reference libraries, databases or, such as HMDB, Compound Discover, METLIN and SMPDB (Xiao et al., 2012). Accurate mass measurements and fragmentation patterns are used to deduce the molecular structure of unknown metabolites.

Metabolites can be quantified either relatively, by comparing the intensity of ion signals between samples, or absolutely, using calibration curves with known standards.

MS is a major platform for clinical metabolomics due to its excellent sensitivity, selectivity, and wide dynamic range (Ding and Feng, 2023). MS-based metabolomics can simultaneously detect and quantify thousands of metabolite features (Alseekh et al., 2021). Common MS-based metabolomics methods include GC-MS and LC-MS. Compared with GC-MS, LC-MS generates extensive data, has high sensitivity, and can measure a wide range of metabolites. Due to the feasibility of liquid chromatography (LC) in separating a wide range of metabolites with broad polarity, combining LC with high-resolution MS systems consistently detects and quantifies thousands of metabolic features, even from minimal sample amounts such as 10 mg of tissue, 50 uL of urine, or as few as half a million cells (Guo et al., 2022a). LC-MS-based metabolomics has gained increasing attention for identifying disease biomarkers and providing unique insights into pathophysiological processes (Ding and Feng, 2023; Randall et al., 2023).

Mass spectrometry imaging (MSI) technology is also widely used in the study of the spatiotemporal distribution of various metabolites, peptides and proteins in animal/plant tissues due to its advantages such as label-free, non-specific, high sensitivity, high chemical coverage, and simultaneous detection of elements/molecules.

### 2.3 Mass spectrometry-based multi-omics applications in thyroid diseases

High-throughput techniques, exemplified by MS, play a crucial role in the measurement of metabolomic and proteomic data (Reel et al., 2021). Collectively, these “-omics” data hold the potential to significantly advance precision medicine, particularly in the context of biomarker-driven approaches for conditions such as endocrine diseases, diabetes, cancer, cardiovascular disease, respiratory disorders, and Alzheimer’s disease (Reel et al., 2021).

Regarding thyroid diseases, understanding the pathogenesis is essential for improving diagnostic accuracy, precise risk stratification, and enabling personalized treatment (Li et al., 2023a). In recent years, with the continuous development of MS, various omics analysis methods based on different sample types (cells, tissues, serum, and urine) have been applied to the study of thyroid disease, actively promoting the development of accurate diagnosis and treatment of thyroid disease by clarifying the pathogenesis, diagnostic grading, prognosis prediction and targeted therapy (Li et al., 2023a).

Biomarkers refer to “an indicator that can be objectively detected and evaluated and can be used as an indicator of normal biological processes, pathological processes, or pharmacological responses to therapeutic intervention” and are of great significance for screening, diagnosing, or monitoring diseases (Biomarkers, 2001; Mischak et al., 2010; Joshi et al., 2024). The exploration of biomarker discovery holds promise in identifying potential markers for early disease detection, prognosis assessment, predicting and monitoring treatment responses (Jimenez and Verheul, 2014). The identification and validation of reliable biomarkers will continue to help improve our understanding of thyroid disease and refine treatment strategies (Davis et al., 2020; Califf, 2018).

Misdiagnosis is common in the diagnosis of thyroid disease (Walsh, 2016). Therefore, it is necessary to identify biomarkers for specific thyroid disease states. MS-based proteomics and metabolomics have been widely used for the discovery of potential biomarkers in the research of thyroid disease.

Much of the published proteomic studies of thyroid disease have compared the protein profiles of thyroid disease groups with healthy thyroid groups to find potential protein markers (Paron et al., 2003). Tissues and cell lines of thyroid are always used for differential proteomics. In a 1997 study, Galectin-3 was proposed to be a potential biomarker of malignant thyroid tumors, especially papillary carcinomas (Fernández et al., 1997). This finding has been confirmed by several other independent researches using different proteomic approaches: MALDI-MSI (Paron et al., 2003), two-dimensional gel electrophoresis and LC-MS (Torres-Cabala et al., 2004). S100 family proteins are comprised of 21 small isoforms, and many of them implicated in important cellular functions such as proliferation, motility and survival (Martinez-Aguilar et al., 2015). Several papers have been published confirming them as potential biomarkers in thyroid cancer by proteomic approaches (Torres-Cabala et al., 2004; Nishimura et al., 1997; Wang et al., 2021). Torres-Cabala, C. et al. identified a new protein, S100C, which is highly expressed in PTC by two-dimensional gel electrophoresis and LC-MS (Torres-Cabala et al., 2004). S100A6 was found to be expressed at a significantly higher level in PTC compared with other tumor groups or normal tissues by LC-MS based proteomics (Sofiadis et al., 2010). Nipp et al. (2012) confirmed S100A10 and S100A6 as biomarkers of PTC with lymph node metastasis identified by MALDI-MSI proteomic approach. This result also demonstrated the potential application of MALDI-MSI proteomic approach in identifying biomarkers in thyroid cancer.

In recent years, exosomes, small membrane microvesicles derived from endosomal cells, have attracted great interest in the proteomics of thyroid diseases due to their role in transporting proteins, lipids and nucleic acids into target cells (Zhang et al., 2019). Transport of molecules via exosomes is one of the factors in the development of thyroid cancer, and the transported molecules can serve as cancer biomarkers (Surman et al., 2024). Luo et al. (2018) compared proteome profiles of serum-purified exosomes (SPEs) from PTC patients with LNM, PTC patients without LNM, and healthy donors. The results showed that specific proteins related to cancer cell metastasis, such as SRC, TLN1, ITGB2, and CAPNS1, were overexpressed in the SPEs of PTC patients with LNM (Luo et al., 2018). In the study of Xi Jia et al., the screened differentially expressed proteins, such as MAP1S, VAMP8, IF5, RSU1, ACTB and CXCL7, were mainly enriched in the immune system and metabolic system that can be seen as potential biomarkers, indicating that plasma exosomes may play an important role in the systemic immune imbalance of autoimmune thyroid diseases (AITDs) (Jia et al., 2021).

Proteomics can not only provide biomarkers for diagnosis but also reveal potential therapeutic targets. For example, protein HSP90 was found to be overexpressed in thyroid cancer (Pearl et al., 2008; Liu et al., 2017). HSP90 regulates protein degradation of several growth-mediating kinases such as BRAF and RET which are well known for the role they play in carcinogenesis (Gild et al., 2016). Several studies have shown that inhibition of HSP90 can not only attenuate cell proliferation but also improve the efficacy of radioiodine therapy in thyroid cancer patients (Gild et al., 2016; Marsee et al., 2004; Wickenberg et al., 2024; White et al., 2016).

Since tumors significantly alter major metabolic pathways, metabolomics is also rapidly becoming an important method for identifying cancer biomarkers. Alterations of the metabolome can be reflected in both tissues and biological fluids. Most chromatography-based metabolomics studies focus on biomarkers between disease and normal groups. Huang et al. (2019a) conducted metabolomic studies using 1,540 clinical serum and plasma samples, along with 114 clinical tissue samples, to characterize the metabolomic profiles of healthy controls and patients with thyroid nodules, including benign thyroid nodules (BTN) and PTC. Their research identified a group of circulating metabolites—myo-inositol, α-N-phenylacetyl-L-glutamine, proline betaine, L-glutamic acid, LysoPC (18:0), and LysoPC (18:1)—as potential biomarkers. Jajin et al. (2022) used GC-MS to perform plasma metabolomics profiling of medullary thyroid cancer (MTC) patients. Results showed that linoleic acid, linolenic acid, and leucine can be used as potential biomarkers for early detection of MTC. These findings provide a basis for the diagnosis and management of thyroid cancer patients from a metabolomics perspective.

Spatially resolved metabolomics integrates MSI and metabolomics technology to accurately measure the types, contents and differential spatial distribution of endogenous or exogenous metabolites in biological tissues and cells and shows great prospect in biomarker discovery of thyroid disease. Jialing Zhang et al. (2017b) used desorption electrospray ionization mass spectrometry imaging (DESI-MSI) to analyze metastatic thyroid cancer in human lymph node tissues and the results showed that the relative abundance of ceramide and glycerophosphoinositide increased.

Wojakowska et al. (2018) used MALDI-MSI to analysis of lipid distribution directly in formalin-fixed tissue. The results showed that the abundance of phosphatidylcholine (32:0, 32:1, 34:1 and 36:3), sphingomyelin (34:1 and 36:1) and phosphatidic acid (36:2 and 36:3) were significantly higher in cancer tissues than them in non-cancer tissues (Wojakowska et al., 2018).

Luojiao Huang et al. (2019b) used the air-flow assisted desorption electrospray ionization (AFADESI) MSI to investigated the metabolic characteristics of different microregions of PTC and results showed that phenylalanine, leucine and tyrosine were expressed at the highest levels in tumors, with a trend of gradually decreasing from tumors to stromal tissues and normal tissues, while creatinine was the opposite.

Biomarker discovery can contribute to molecular subtyping in thyroid disease. The integration of MS-based multi-omics is a powerful tool for elucidating complex molecular signatures of various cancer subtypes (Wang et al., 2023b). This approach not only enhances our understanding of the mechanisms of action of various molecules within cancer but also facilitates more targeted and personalized therapeutic interventions for specific subtypes (Berger and Mardis, 2018).

Martinez-Aguilar et al. (2016) applied DIA MS for quantitative analysis of expression levels for over 1,600 proteins across 32 specimens, discerning differences between normal thyroid tissue and the three prevalent thyroid gland tumors: follicular adenoma, follicular carcinoma, and papillary carcinoma. Proteomic pathway analysis revealed that changes in papillary carcinomas are associated with disruption of cell contacts (loss of E-cadherin), actin cytoskeletal dynamics, and loss of differentiation markers, characteristics of the aggressive phenotype (Martinez-Aguilar et al., 2016).

Wojakowska et al. (2015) used the GC-MS method to extract, identify, and semi-quantitate metabolites in formalin-fixed paraffin-embedded (FFPE) tissue specimens from five different types of thyroid malignancies, benign follicular adenoma and normal thyroid and concluded that multicomponent metabolomic signatures can be used to classify different subtypes of follicular thyroid lesions.

MS-based multi-omics have significantly increased in recent years and enabled mapping of biochemical changes in thyroid disease and hence can provide an opportunity to develop predictive biomarkers that can trigger earlier interventions (Wang et al., 2013). MS can directly quantify thyroid analytes and its high resolution can enhance the accuracy and detection (Jasem et al., 2024). Biomarkers of thyroid disease screened out by MS-based proteomics and metabolomics can not only provide a basis for clinical diagnosis, but also provide insights into the biological mechanisms of thyroid disease. It can be used to distinguish different types of thyroid cancer, which is beneficial for classifying benign and malignant cancers for treatment, such as using different dosing strategies, thereby achieving precision medicine.

## 3 Classic machine learning models and multi-omics applications in thyroid disease

This section will present several classic machine learning models and offer an overview of how these models contribute to the study of thyroid diseases.

### 3.1 Classic machine learning models in data analysis

Machine learning (ML) is the science of developing algorithms and statistical models, which is a subset of artificial intelligence (Smith et al., 2023). Computer systems utilize these algorithms and models to perform tasks without explicit instructions, enabling machines to undertake activities requiring human intelligence, such as diagnosis, planning, and prediction, based on established patterns and reasoning (Mohammadzadeh et al., 2024). In recent years, the exponential growth of biomedical data has driven many applications of ML techniques to address new challenges in biology and clinical research (Auslander et al., 2021). ML methods are favored in statistical analysis because of their inherent nonlinear data representation and ability to quickly process large datasets (Liebal et al., 2020). ML algorithms are employed for training, key feature identification, and group classification (Huang et al., 2018).

**FIGURE 3:**
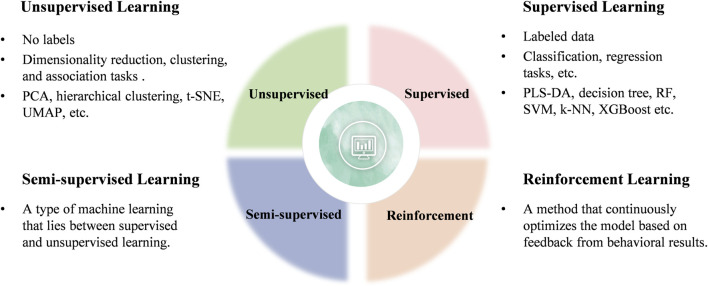
Classification of ML learning algorithms.

Generally, ML can be categorized into four main types: unsupervised, supervised, semi-supervised, and reinforcement learning (Figure 3). Current research in clinical diseases predominantly focuses on unsupervised and supervised learning algorithms, which will be the focus of this review (Perakakis et al., 2018).

This section examines the role of ML in handling and analyzing the vast and complex datasets generated by MS-based multi-omics approaches. It discusses specific algorithms and techniques for data processing, feature selection, and classification, emphasizing their importance in identifying potential biomarkers and therapeutic targets.

#### 3.1.1 Unsupervised learning

Unsupervised learning involves using datasets that contain only input data and attempts to find structure in the data by grouping or clustering the data points (Angra and Ahuja, 2017). Unsupervised learning algorithms are primarily employed for dimensionality reduction, clustering, and association tasks (Arjmand et al., 2022). Four common unsupervised algorithms include principal component analysis (PCA), t-distributed stochastic neighbor embedding (t-SNE), uniform manifold approximation and projection (UMAP), and hierarchical clustering (HCL).

##### 3.1.1.1 Principal component analysis (PCA)

The complexity of multivariate data often necessitates the use of dimensionality reduction methods to simplify the information. Dimensionality reduction of high-dimensional data plays a crucial role in downstream tasks such as pattern recognition, classification, and clustering (Kim et al., 2018). Principal component analysis (PCA) is a classic unsupervised dimensionality reduction method that identifies hidden features in data, providing the most significant signals, and is often used in machine learning. (Kim et al., 2018; Chen and Gao, 2016; Ma and Dai, 2011). PCA simplifies complex data and makes the analysis process easier. Essentially, PCA is an “unsupervised” method that analyzes data purely based on its characteristics, without knowing the grouping of each sample. PCA effectively identifies the “main” elements and structures in the data, removes noise and redundancy, reduces the dimensionality of complex data, and reveals the simple structure hidden behind the complex data (Sugimoto et al., 2012). PCA is widely used in MS-based multi-omics data analysis, particularly for data dimensionality and achieving data visualization (Sugimoto et al., 2012). The results are often visualized using scatter plots.

PCA is commonly applied in clinical analysis to reveal differences between samples, with the distance between samples on the horizontal and vertical axes representing the similarity distance of the samples under the influence of the principal components (PC1 and PC2).

For example, in a single-cell proteomics study of hepatocytes by Rosenberger et al. (2023), PCA was used to reduce the dimensionality of proteomics data, resulting in clear hepatocyte partitioning and demonstrating the biological validity of the data. Similarly, Xu et al. (2022) applied PCA to validate the metabolic profile of mouse liver tissue in a study on the mechanism of action of Huang Qin decoction for treating diabetic liver injury.

PCA is particularly suitable for initial exploratory data analysis, especially when linear relationships are presumed in the data and the interpretability of components is crucial (Beattie and Esmonde-White, 2021; Ivosev et al., 2008). It is also preferred in scenarios where computational efficiency is a priority. However, PCA may not adequately capture complex nonlinear interactions present in biological data.

##### 3.1.1.2 t-distributed stochastic neighbor embedding (t-SNE)

t-distributed Stochastic Neighbor Embedding (t-SNE) is a nonlinear dimensionality reduction technique primarily designed for data visualization and excels at identifying and discovering complex nonlinear structures in data (Cieslak et al., 2020). It converts high-dimensional data into a low-dimensional space, typically two or three dimensions while preserving the local structure of the data (Cieslak et al., 2020; Da Silva Lopes et al., 2020). t-SNE focuses on preserving the relative distances between similar data points, making it effective for revealing clusters and patterns (Chatzimparmpas et al., 2020).

In the study by Liang et al. (2022), 1,681 proteins were analyzed through proteomics in 258 HCM patients. t-SNE was utilized to visualize and reduce the dimensionality of the data, revealing that the four molecular subtypes were well separated.

t-SNE is ideal for visualizing small to medium-sized datasets where the primary goal is to understand and explore local structures and clustering. It is particularly suitable for high-dimensional data with complex, nonlinear relationships (Gisbrecht et al., 2015). However, it is relatively slow, especially when applied to large datasets.

##### 3.1.1.3 Uniform manifold approximation and projection (UMAP)

Uniform manifold approximation and projection (UMAP), an algorithm developed by McInnes et al., is a nonlinear dimensionality reduction technique (Lundberg and Lee, 2017). It is another nonlinear dimensionality reduction method particularly effective at preserving both local and some global structures of the data (Becht et al., 2018; Yang et al., 2021). UMAP is based on manifold learning techniques and constructs a high-dimensional graph representation of the data, which is subsequently optimized to create a low-dimensional embedding (McInnes et al., 2018).

Like t-SNE, UMAP is also effective in capturing complex nonlinear relationships in the data. In general, UMAP is faster and more scalable than t-SNE and it better preserves both local and global structures.

UMAP is preferred for large datasets requiring a balance between local and global structure preservation (Sainburg et al., 2021). It is effective for visualizing complex data structures and works well for high-dimensional data (Becht et al., 2018). Compared to PCA, this method is more complex and can be challenging to understand and debug. Although UMAP is faster than t-SNE, it still requires significant computational resources for very large datasets (Roca et al., 2023).

##### 3.1.1.4 Hierarchical clustering (HCL)

A crucial component of unsupervised learning is the clustering algorithm. Traditionally, cluster analysis is classified as unsupervised learning because it does not involve class labels or quantitative response variables, which are characteristic of supervised learning methods such as classification and regression (Pan et al., 2013). Cluster analysis is a series of different algorithms that divide observation data into different categories or clusters based on distance functions (Blekherman et al., 2011). The goal is to partition the data into groups such that the distance between samples within each group is smaller than the distance between samples in different groups (Blekherman et al., 2011). Hierarchical clustering (HCL) is a clustering method frequently used in marker screening and enables visualization of gene, protein, and metabolite features (Picard et al., 2023; Granato et al., 2018). The results of HCL are commonly visualized using heatmaps.

In biomedical informatics, HCL is often applied to cluster protein sequence data. Proteins with similar structures also have similar functions. Proteins with similar functions can be grouped into categories through clustering, aiding in the study of protein functions. In clinical analysis, hierarchical clustering is utilized to intuitively display relationships between groups and highlights expression differences of characteristic substances.

In the study of MS-based urine proteomics of gastric lesions by Fan et al. (2022), HCL was used to partition 139 differential proteins with VIP>1 into six clusters, revealing dynamic changes from precancerous lesions dynamic changes in gastric cancer.

In an MS-based metabolomics study of cancer cell lines by Li et al. (2019), HCL was employed to assess metabolic similarities between cell lines.

For MS-based spatial proteomics, dimensionality reduction and clustering methods such as PCA, t-SNE and HCL are effective for quality control of MS-based spatial proteomic data and for examining organelle separation (Huang et al., 2022; Karimpour-Fard et al., 2015; Mou et al., 2022; Ringner, 2008).

#### 3.1.2 Supervised learning

Supervised learning relies on labeled datasets to train algorithms on a predefined classification system and to infer the functional relationship between input features and output labels based on this training. The algorithm learns a function from the training dataset that enables prediction of outcomes for new data. In essence, supervised learning involves determining whether the objective is to predict outcomes based on known input-output pairs. A significant category within supervised learning is classification problems. In the classification problems, the target variables are discrete rather than continuous. Examples include tumor size, patient age, and the benign or malignant status of the tumor.

The process of model training with ML algorithms involves three steps: data splitting, parameter estimation using the training set, and performance evaluation using the test set.

a. Data Splitting

Typically, the dataset used to train a ML model is divided into a training set and a test set, with a common ratio of 70:30 (Galal et al., 2022). A validation set is often included for model performance evaluation and hyperparameter tuning, ensuring optimal results under the given data conditions. In this scenario, the data can be divided into 60% training, 20% validation, and 20% test sets (Galal et al., 2022).

b. Parameter Estimation Using Training Data

Parameter estimation is a critical step in model training. The goal of using training set to estimate model parameters is to create a model that accurately captures the underlying information in the data so that it can make reliable predictions about new data.

c. Comprehensive Evaluation Using Test Data

The test set evaluates the overall performance of the final model. After final parameter adjustments, the test set is used to evaluate the performance of the model comprehensively, assessing for issues such as overfitting or underfitting. If no issues are identified, the model can be applied to the project.

Common supervised algorithms include partial least squares discriminant analysis (PLS-DA), decision trees (DTs), random forest (RF), support vector machine (SVM), K-Nearest Neighbor (kN), and eXtreme Gradient Boosting (XGBoost).

##### 3.1.2.1 partial least squares discriminant analysis (PLS-DA)

Partial least squares discriminant analysis (PLS-DA) is also a dimensionality reduction algorithm. Unlike PCA, PLS is a “supervised” mode of partial least squares analysis, meaning that the grouping relationships of the samples are known, allowing for better selection of characteristic variables that distinguish each group and determination of relationships between the samples. DA stands for discriminant analysis. PLS-DA employs the partial least squares regression method to “reduce the dimensionality” of the data, establish a regression model, and conduct discriminant analysis on the regression results. PLS-DA is particularly suitable for selecting and interpreting metabolite signatures when studying biological systems (D'Andrea et al., 2023).

In an MS-based urine proteomics study of gastric lesions by Fan et al. (2022), PLS-DA was used to analyze the proteomics data of different groups and screen out 139 differential proteins with VIP>1.

In the study by D'Andrea et al. (2023), the PLS-DA model was confirmed through cross-validation, and the average variable importance in projection (VIP) score was used to identify metabolites that differed among sample classes.

##### 3.1.2.2 Decision trees (DTs)

Decision trees (DTs) employ ML techniques to address classification and prediction problems. Nodes and leaves are the primary elements that form a decision tree (Chaubey et al., 2020). Nodes test specific properties, and leaves represent a class (Mesarić and Šebalj, 2016). Common decision trees include the ID3 tree, the C4.5 tree (information gain rate), and the CART tree (Gini coefficient) (Ross, 1993; Leo et al., 1984).

The ID3 algorithm is one of the classic decision tree algorithms. The C4.5 algorithm is an improvement upon the ID3 algorithm and can handle discontinuous features (Navada et al., 2011). The ID3 and the C4.5 algorithm are primarily used to address classification problems, but cannot be used to apply regression problems (Singh and Giri, 2014). The CART algorithm can manage both classification and regression problems.

Fannes et al. (2013) introduced CP-DT (Decision Tree Cleavage Prediction), an algorithm based on an ensemble of decision trees trained on publicly available peptide identification data from the PRIDE database. The study demonstrated that CP-DT can accurately predict trypsin cleavage (Fannes et al., 2013).

Decision tree algorithms are fast, however, they are generally not as accurate as other models.

##### 3.1.2.3 Random forest (RF)

Random forest (RF) is a regression tree technique that employs bootstrap aggregation and predictor randomization to achieve a degree of predictive accuracy (Steven, 2017). Proposed by Breiman in 2001, RF employs randomization to create numerous decision trees and is a widely used tool for classification and regression in bioinformatics and related fields (Steven, 2017; Janitza et al., 2016). Compared to a single decision tree, a random forest exhibits stronger generalization performance. In classification problems, the outputs of these decision trees are voted and aggregated into one output; in regression problems, they are averaged and aggregated into one output (Steven, 2017). RF classification is a widely used supervised learning method for developing predictive models in many research settings (Speiser et al., 2019).

The random forest algorithm is simple and easy to implement, applicable to both classification and regression problems (Steven, 2017). It has the following features.

a. It can handle numerous input variables, and the more data features present, the more stable the model (Belgiu and Drăguţ, 2016).
b. It can evaluate feature importance while determining the category (Archer and Kimes, 2008; Khalilia et al., 2011).
c. It can estimate valuable data and maintain a certain degree of accuracy even when a significant portion of the data is missing.

Khalilia et al. (2011) utilized National Inpatient Sample (NIS) data from the Healthcare Cost and Utilization Project (HCUP) to train RF classifiers for predicting eight disease categories. The results demonstrated good performance (Khalilia et al., 2011).

However, RF does not perform as well for regression problems as it does for classification and may not produce good classification results for small or low-dimensional datasets (datasets with fewer features).

##### 3.1.2.4 Support vector machine (SVM)

Support vector machine (SVM) is a supervised algorithm that learns from examples to assign labels to objects (Boser et al., 1992). Compared to other ML methods, SVM is highly effective at identifying subtle patterns in complex data sets (Aruna and SP, 2011). The purpose of SVM is to create a decision boundary between two categories, facilitating the prediction of a label based on one or more feature vectors (WS, 2006). This decision boundary, called a hyperplane, should be oriented as far away as possible from the nearest data point for each class, referred to a support vector (Huang et al., 2018).

The computational complexity of SVM depends on the number of support vectors rather than the dimension of the sample space, thereby avoiding the “curse of dimensionality” (Markowetz, 2001). However, SVM is sensitive to missing data and is difficult for solving multi-classification problems (Cervantes et al., 2020). In areas where SVM performs poorly, researchers have developed other applications such as SVM for large datasets, multiple classifications, and imbalanced datasets (Cervantes et al., 2020).

Mavrogeorgis et al. (2023) obtained urine peptide data of 1850 healthy controls (HC) and CKD (diabetic nephropathy-DKD, IgA nephropathy-IgAN, vasculitis) participants from the Human Urine Proteome Database. UMAP was combined with SVM for binary (DKD, HC) and multi-class (DKD, HC, IgAN, vasculitis) classification.

##### 3.1.2.5 K-Nearest neighbor (k-NN)

K-Nearest Neighbor (k-NN) is a simple and practical supervised learning algorithm frequently used to deal with classification problems (Boateng et al., 2020). It examines the k nearest sample points closest to the new sample point in the training set, using a specific distance metric, and classifies the new sample point into the category with the most occurrences among the k sample points (Abu Alfeilat et al., 2019). The parameter k is crucial, and its value should be optimally chosen (Zhang et al., 2017a). A value that is too low will increase the error rate, while a value that is too high can render the model ineffective (Zhang et al., 2017b).

The algorithm is simple in principle, easy to understand and implement, applicable to multi-classification problems, and requires no additional processing (Chaubey et al., 2020). However, k-NN involves substantial computational effort and requires considerable memory resources. Its performance is influenced by the parameter k and it tends to perform poorly on unbalanced datasets.

##### 3.1.2.6 eXtreme gradient boosting (XGBoost)

eXtreme Gradient Boosting (XGBoost) is a machine learning model built on a decision tree ensemble and is among the most widely used machine learning algorithms (Kavzoglu and Teke, 2022). The algorithm has the following features:

a. It excels in processing both structured and unstructured data, frequently achieving higher accuracy compared to other algorithms (Arif Ali et al., 2023).
b. It relies on on decision tree integration, offers excellent interpretability, and provides insights into the importance of each feature (Kavzoglu and Teke, 2022).
c. It employs parallel computing technology and demonstrates high computational efficiency in processing large-scale data (Nalluri et al., 2020).

Li et al. (2024) developed a model incorporating 17 feature variables using XGBoost, based on the multidimensional data from a retrospective cohort of 274 papillary thyroid carcinoma (PTC) patients. This model demonstrated strong predictive performance in differentiating between low-risk and medium/high-risk PTC cases and was designated as the PTC Preoperative Risk Assessment Classifier (PRAC-PTC).

However, it is sensitive to parameters settings, with the choice of parameters significantly influencing the results (Demir and Şahin, 2022). In some cases, XGBoost may be overly complex and prone to overfitting the training data.

### 3.2 Applications of machine learning in MS-based multi-omics in thyroid disease

#### 3.2.1 Applications in MS-based multi-omics data analysis

Extracting valuable insights from MS-based multi-omics data presents a significant challenge in bioinformatics (Tang et al., 2019). The complexity and high dimensionality of MS-based multi-omics datasets make traditional analysis methods challenging (Krassowski et al., 2020). Combining ML methods with MS-based multi-omics analysis mainly involves integrating various ML techniques to manage the complexity and volume of multi-omics data, aiming to enhance both accuracy and interpretability.

##### 3.2.1.1 Missing data imputation

Missing data refers to the situation where data is incomplete due to some reasons during the process of data collection, transmission, and processing (Du et al., 2020). It is a common problem in MS-based omics data analysis (Huang et al., 2023). The simplest way to deal with missing values is to remove samples with missing values. However, if there are many missing values, such as the missing data of LC-MS-based omics data may be in the range of 30%–50%, a large number of samples will be eliminated, resulting in the loss of more useful information (Liebal et al., 2020).

Imputation methods provide an alternative way of handling missing data rather than discarding missing values and associated data (Huang et al., 2023). The mean, median, mode, etc., of the feature can be used to fill the missing values (Emmanuel et al., 2021). However, these simple methods do not consider the relationship between data variables, which sometimes makes the results of data analysis unreliable. Among the methods for dealing with missing values, many other filling methods consider the relationship between data variables (Baraldi and Enders, 2010). ML algorithms, such as regression, k-NN, and RF, can help resolve missing data problems in multi-omics datasets by inferring values based on observed patterns in existing data (Emmanuel et al., 2021; Mirza et al., 2019).

##### 3.2.1.2 Dimensionality reduction

MS-based multi-omics data may have multiple layers of variables and a large number of attributes, so-called high-dimensional data (Arjmand et al., 2022). While high-dimensional data will cause great trouble for subsequent data processing (Cao and Lin, 2015), dimensionality reduction is a crucial step (Fanaee and Thoresen, 2019). It aims to reduce the number of variables considered, making the data more manageable and easier to analyze while retaining as much information as possible. Before applying dimensionality reduction, multi-omics data need to be preprocessed, such as normalization and missing value filling, to ensure that the data is in a form suitable for analysis (Reska et al., 2021). Dimensionality reduction improves computational efficiency, reduces noise while retaining important information, facilitating data processing (Alhassan and Wan Zainon, 2021). Many ML algorithms can facilitate data processing by reducing data dimensionality while retaining important information, such as PCA, t-SNE, PLS-DA, and UMAP.

##### 3.2.1.3 Clustering and classification

Clustering is an unsupervised learning method that groups data based on the attributes of the input features (Reel et al., 2021). Classification is a supervised learning method that provides predicted output as a discrete class (Reel et al., 2021). ML algorithms can group samples into clusters or classify them based on distinct patterns present in multi-omics data which can discover subtypes or stratify patients and identify similarities among clustered patients (Goecks et al., 2020).

##### 3.2.1.4 Feature selection

Feature selection is a key step in multi-omics analysis and helps reduce data dimensionality. In this sense, feature selection has similar motivations to dimensionality reduction as described above. Feature selection can remove irrelevant features and reduce the number of features used in the analysis, thereby reducing the difficulty of the learning task (Li et al., 2017a). It should be noted that the feature selection process must ensure that no important features are lost.

For a multi-omics dataset, a set of attributes is included, some of them may be critical and useful, while others may be useless. Attributes are called features, those that are useful for the current learning task are called relevant features, and those that are useless are called irrelevant features (Kotsiantis, 2011). The process of selecting a subset of relevant features from a given set of features is called feature selection (Jović et al., 2015).

Feature selection is an important data preprocessing process. In real machine learning tasks, feature selection is usually performed after obtaining multi-omics data, and then the learner is trained.

In practical applications, feature selection methods are mainly divided into filter, wrapper, and embedded methods (Venkatesh and Anuradha, 2019).

a. Filter Selection

Filter selection first selects features from the data set and then trains the learner (Li et al., 2017a). The feature selection process is independent of the subsequent learner. It is the simplest and most commonly used method to implement feature selection. The core of the filtering method selection is to sort the features according to their value, to achieve the selection or elimination of any proportion/quantity of features.

Filter selection is computationally efficient and relatively simple to implement, but it ignores the interactions between features (Jiliang et al., 2014).

b. Wrapper Selection

Unlike filter-based feature selection, which does not consider subsequent learners, wrapper selection directly uses the performance of the most important learner as the evaluation criterion for the feature subset (Wald et al., 2013; Cadenas et al., 2013).

Since wrapper selection directly optimizes a given learner, it is better than filter selection in terms of the final learner performance (Cadenas et al., 2013). However, since the learner needs to be trained multiple times during the feature selection process, it requires a huge amount of computation.

c. Embedded selection

Embedded selection integrates the feature selection process with the learner training process, that is, feature selection is automatically performed during the learner training process (Li et al., 2017b). The most commonly used are tree models and a series of ensemble algorithms based on tree models because the model provides important information about feature importance.

Embedded selection combines the advantages of filter selection and wrapper selection in terms of computational cost and performance, but it cannot identify highly relevant features (Remeseiro and Bolon-Canedo, 2019).

#### 3.2.2 Applications in clinical researches

The contribution of ML to thyroid disease is not only in processing data, but also in the classification, clinical diagnosis, treatment, prognosis and risk stratification of thyroid diseases. In clinical researches, ML is beneficial and essential (Komuro et al., 2023). ML enables efficient analysis of extensive data sets and improve disease diagnosis and classification by building predictive models of disease, and has a particularly important impact on improving MS-based clinical multi-omics research (Perakakis et al., 2018; Arjmand et al., 2022). ML models primarily select important features from complex data to construct predictive models and output data as predictive labels based on identified patterns (Ngan et al., 2023). Using models like decision trees, RF, SVM, and XGBoost to predict outcomes based on data facilitates predictive models, including disease diagnosis, prognosis, and treatment, based on multi-omics data (Quazi, 2022; Wekesa and Kimwele, 2023). Validation and evaluation are critical steps in ML for multi-omics analysis to ensure that the models and methods used are reliable and robust. There are several approaches:

Cross-Validation: Use k-fold cross-validation to assess model performance.

External Validation: Validate results with independent datasets to ensure robustness.

Performance Metrics: Use appropriate metrics to evaluate the model (e.g., accuracy, precision, recall, area under curve (AUC), F1-score for classification; R 2, RMSE for regression).

The trained and validated ML models are expected to be able to assess cancer risk and facilitate the development of preoperative cancer diagnosis (Ngan et al., 2023).

##### 3.2.2.1 Diagnosis and classification

**FIGURE 4:**
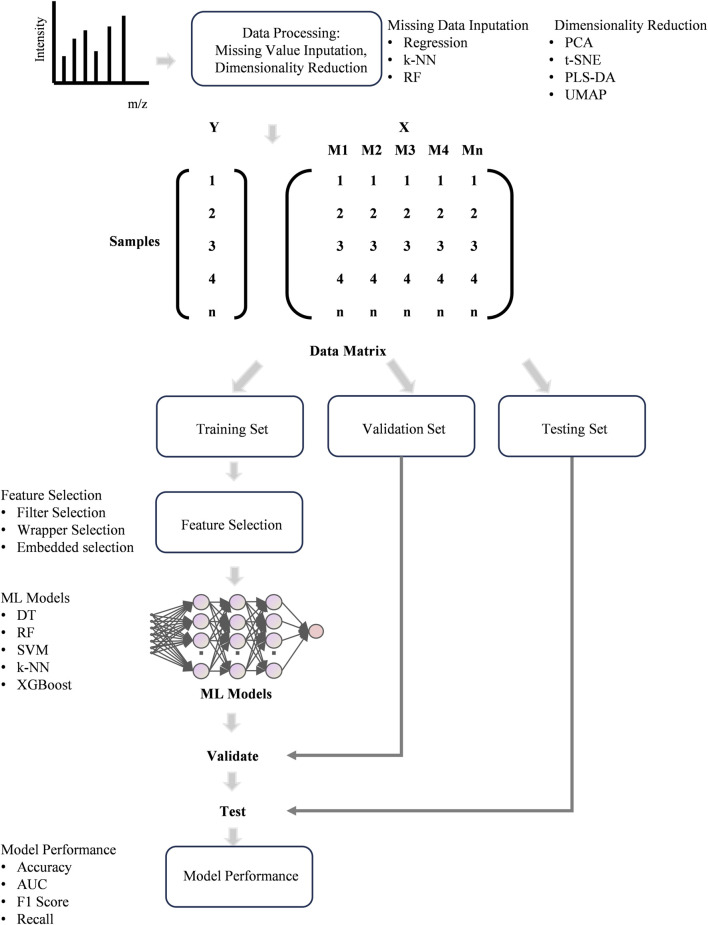
The workflow of ML for data processing and modeling.

The classification model is the most commonly used type of ML models in clinical researches which can output the type of thyroid disease it predicts based on the input features, such as clinical factors and omics data, thereby providing insights into clinical diagnosis (Figure 4). For thyroid disease prediction, ML methods have been applied in various existing research works. Prediction of thyroid disease at its early stages and categorization into cancer or other thyroid disease is very helpful for treating and recovering the maximum number of patients (Gupta et al., 2024). MS-based multi-omics have become a powerful technique for biomarker discovery which is significant for clinic (Torun et al., 2022). However, screening biomarkers from complex MS data requires reliable bioinformatics tools and ML can be a good choice (Torun et al., 2022). Analyzing large and complex datasets by ML models enables the identification of subtle biomarkers and disease signatures, which leads to earlier and more accurate diagnoses (Ng et al., 2023). This is particularly beneficial in complex diseases specially cancer, where early detection can significantly improve prognosis. The integration of ML and MS-based multi-omics not only improves the efficiency of biomarker discovery but also helps develop more accurate and reliable diagnostic tools, ultimately advancing the field of precision medicine and improving patient outcomes (Johnson et al., 2021; Krishnan et al., 2023).

From the perspective of classification, existing researches mainly focused on binary classification problems in thyroid disease classification based on ML models. Kumari et al. (2024) used age, gender, and hormone levels as features and combined them with 3 ML models to classify hyperthyroidism and hypothyroidism. Results showed that XGBoost was the top-performing model for this task (Kumari et al., 2024).

Al-muwaffaq and Bozkus (2016) developed the Machine Learning tool for Thyroid Disease Diagnosis (MLTDD) mainly focused on thyroid gland medical diseases caused by underactive or overactive thyroid glands. The prediction accuracy was in range between 98.7% and 99.8% for testing. MLTDD can effectively help to make the right clinical decision.

Xi et al. (2022) constructed 6 ML models to predict the malignancy of thyroid nodules. RF and Gradient Boosting Machine (GBM) showed better overall diagnostic accuracy and ability to identify malignant nodules. Their method can be used as additional evidence in the preoperative diagnosis of thyroid cancer.

Guo et al. (2022b) constructed a diagnostic model of benign and malignant thyroid tumors. The benign group contained 5 thyroid diseases and the malignant group contained six thyroid diseases. Clinical factors were used as features and RF, XGBoost, LightGBM, and AdaBoost models were constructed. RF model showed the best performance. Their research proposed a model incorporating novel biomarkers which could be a powerful and promising tool for predicting benign and malignant thyroid tumors (Guo et al., 2022b).

Recently, some researches have begun to focus on multi-classes classification problems. Chaganti et al. (2022) used ML models to predict five thyroid diseases. Results showed that the extra tree classifier-based selected feature yields the best results with 0.99 accuracy and an F1 score when used with the RF classifier (Chaganti et al., 2022).

Gupta et al. (2024) used ML models to predict ten thyroid diseases. Results suggested that an accuracy of 0.998 can be obtained using the optimized AdaBoost model by differential evolution (Gupta et al., 2024). These researches applied ML models to a variety of thyroid diseases, which helped to diagnose diseases more conveniently and comprehensively in clinical practice.

ML models can not only be used to classify the types of thyroid disease, but also to determine whether there is a certain gene mutation in the disease. Kwon et al. (2020) used ML models combined with radiomics to predict the presence or absence of B-Raf proto-oncogene, serine/threonine kinase (BRAF) mutation in PTC and the results showed that the classification accuracy of these models was higher than 60%. Although this research provided a new perspective of application of ML in thyroid disease which focused on the gene mutation, the classification model did not show excellent performance in predicting the presence of BRAF mutation in PTC and need to be further validated in a larger dataset to better assess their potential clinical use.

In addition, ML models can also be used to predict treatment trends and prognosis. Aversano et al. (2021) combined ten different ML models with parameters related to the person being treated to predict whether the LT4-based treatment needs to be increased or decreased for the patients with hypothyroidism. This study provides reference insights for clinical treatment plans.

Wang et al. (2024a) presented ML models based on comprehensive predictors to predict the structural recurrence risk of PTC patients. All the patients were treated with thyroid surgery and radioiodine. Twenty-nine perioperative variables consisting of four dimensions (demographic characteristics and comorbidities, tumor-related variables, lymph node-related variables, and metabolic and inflammatory markers) were analyzed (Wang et al., 2024a). The results showed that the RF model achieved the expected prediction effect, with good discrimination, calibration, and interpretability, and revealed the potential of ML models in improving the accuracy of risk stratification of PTC patients (Wang et al., 2024a).

It is also an important task for ML models to classify clinical patient samples based on MS-based multi-omics data. In many cases, it can be used to determine which ion m/z values contribute most to the models, thereby facilitating the identification of biomarkers and further facilitating the diagnosis and classification of thyroid disease (Beck et al., 2024). Recently, an increasing number of researches have focused on combining ML with MS data for thyroid disease classification, even for further treatment strategies, which have provided broader prospect.

Sun et al. (2022) proposed the first protein-based neural network classifier for thyroid nodules. Their research was the first to establish a deep proteome data repository for various thyroid lesions, analyzed a larger sample size and obtained deeper proteome coverage (Sun et al., 2022). The large-scale thyroid proteome map combined with the neural network model demonstrated the power of the classifier and is expected to be quickly applied to clinical practice to supplement the deficiencies of traditional cytopathology (Sun et al., 2022). Their recent work (Sun et al., 2024) established a protein-based model with targeted MS for the diagnosis of FTC and FTA. This model used 24 proteins filtered by XGBoost as features and performed better than gene-based model. The protein model has 95.7% negative predictive value for ruling out malignant nodules (Sun et al., 2024).

Wang et al. (2022b) develop a rapid classification method by ML and MS-based metabolomics to diagnose PTC. The metabolomics of frozen samples were performed by probe electrospray ionization (PESI) mass and SVM and RF models were used. For the classification of PTC from PTC adjacent tissues, SVM performed better than RF. Their another work developed a rapid method to classify the malignant and benign thyroid nodules by PESI-MS-based machine learning (Wang et al., 2022c). For each FNAB sample, only 10 min is needed to determine its malignancy, which is much easier and faster than traditional diagnosis (Wang et al., 2022c).

Chen et al. (2024) explored the potential of rapid thyroid disease screening using the ZrMOF/Au-assisted LDI-MS platform, enabling rapid screening of malignant thyroid disease from benign patients. The authors constructed a panel of 43 key metabolites as features for ML models to discriminate thyroid cancer from thyroid nodules and NN, RF, LR, and SVM models were used for classification (Chen et al., 2024). The results showed that NN had the best classification performance.

Zhu et al. (2024) combined ML with MS-based proteomics and selected four proteins as features with the highest contributions to predict the efficacy of iodine therapy. This research showed the ability to pre-identify PTC patients who are resistant to radioactive iodine therapy (Zhu et al., 2024).

The above articles were retrieved by entering “machine learning” and “thyroid disease” in Google Scholar and some representative ones involving “application of ML in diagnosis of thyroid disease” were selected by us. We showed them in Table 1 in the order we mentioned in this section with three summary measures, sample types, features, and supervised ML models.

**TABLE 1:** Applications of machine learning in diagnosis of thyroid disease.

References	Year	Sample types	Features	Supervised ML models
Gupta et al. (2024)	2024	Concurrent Non-thyroid Illness, Compensated Hypothyroid, Increased Binding Protein, Primary Hypothyroid, etc	not given	RF, SVM, LR, ADA, GBM, CNN, RNN, LSTM
Kumari et al. (2024)	2024	Hyperthyroidism and Hypothyroidism	age, sex, pregnancy, T3, T4, and TSH	RF, SVM, XGB
Al-muwaffaq and Bozkus (2016)	2016	Hyperthyroidism, Hypothyroidism and normal function of the thyroid gland	age, sex, on_thyroxine, query_on_thyroxine, on_antithyroid_medication, sick, pregnant, TSH, T3, T4, T4U, FTIetc.	DT
Xi et al. (2022)	2022	Thyroid Cancer	demographic information, ultrasound features, and blood test results	RF, SVM, LR, LDA, GBM
Guo et al. (2022b)	2022	Benign and Malignant Thyroid Tumors	Peripheral blood indicators, BRAFV600E gene, demographic indicators	RF, XGB, GBM, ADA
Chaganti et al. (2022)	2022	Hashimoto’s thyroiditis, binding protein (increased binding protein), Autoimmune Thyroiditis, and Non-Thyroidal Syndrome (NTIS)	age, sex, on_thyroxine, query_on_thyroxine, on_antithyroid_medication, sick, pregnant, TSH, T3, T4, T4U, FTI, TBGetc.	RF, LR, SVM, ADA, GBM
Kwon et al. (2020)	2020	PTC	radiomics features	LR, RF, SVM
Aversano et al. (2021)	2021	Hypothyroidism	personal information, family history, physical characteristics, hormonal and thyroid parameters, parameters relating to blood tests	ADA, XGB, GBM, CAT, DT, RF, ExtraTree, k-NN, Naive Bayes, MLP
Wang et al. (2024a)	2024	PTC	demographic characteristics and comorbidities, tumor-related variables, lymph node (LN)-related variables, metabolic and inflammatory markers	LR, XGB, RF, SVM, NN
Sun et al. (2022)	2022	Thyroid Nodules	MS-based proteomics	NN
Sun et al. (2024)	2024	FTA and FTC	MS-based proteomics	XGB
Wang et al. (2022b)	2022	PTC	MS-based metabolomics	RF, SVM
Wang et al. (2022c)	2022	Thyroid Nodules	MS-based ions	RF, SVM, MLP
Chen et al. (2024)	2024	Thyroid Cancer and Thyroid Nodules	MS-based metabolomics	NN, RF, LR, SVM
Zhu et al. (2024)	2024	Radioactive Iodine Refractory (RAIR) and Non-Radioactive Iodine Refractory (Non-RAIR) PTC	MS-based proteomics	XGB

ADA, adaptive boosting; CAT, CatBoosting; CNN, convolutional neural network; DT, decision tree; GBM, gradient boosting machine; k-NN, k-nearest neighbor; LDA, linear discriminant analysis; LR, logistic regression; LSTM, long short-term memory; MLP, multilayer perceptron; NN, neural network; RF, random forest; RNN, recurrent neural network; SVM, support vector machine; XGB, eXtreme Gradient Boosting.

By analyzing the above research works, we found that patients’ personal data and hormone parameters are often used as features, such as age, sex, thyroid-stimulating hormone (TSH), total serum triiodothyronine (T3), thyroid binding globulin (TBG) and total serum thyroxin (T4). Among these features, almost every researcher has selected some features for thyroid disease diagnosis work. MS-based proteomics and metabolomics data are also often used but almost only single type is used. In order to analyze thyroid disease more comprehensively, combining metabolomics and proteomics or combining them with other omics data such as genomics and clinical indicators should be further considered in future studies.

##### 3.2.2.2 Risk stratification

A significant advantage of ML in the clinic is its ability to facilitate early intervention (Adlung et al., 2021). By using advanced algorithms and predictive models, ML algorithms can detect potential health risks at an early stage, and effectively classify diseases into different risk categories, which offer a nuanced understanding of the likelihood of patients developing specific health conditions and allowing clinicians to intervene promptly and implement targeted treatments (Beaulieu-Jones et al., 2021; Choudhury and Asan, 2020; Sun et al., 2023). This goes beyond traditional diagnostic methods, providing a more personalized and proactive approach to healthcare (Yadav, 2024).

Saima Sharleen Islam (Sun et al., 2023) et al. used 11 ML models to predict thyroid risk and used accuracy and recall as evaluation indicators. The results show that the ANN classifier outperforms the others in terms of accuracy. Zhao et al. (2022a) et al. used 5 ML models to predict the risk of nodular thyroid disease in coal miners, with the XGB model having the best overall predictive performance.

Wang et al. (2022a) presented ML models based on comprehensive predictors to predict the structural recurrence risk of PTC patients. All the patients were treated with thyroid surgery and radioiodine. Twenty-nine perioperative variables consisting of four dimensions (demographic characteristics and comorbidities, tumor-related variables, lymph node-related variables, and metabolic and inflammatory markers) were analyzed Wang et al. (2022a). The results showed that the RF model achieved the expected prediction effect, with good discrimination, calibration, and interpretability, and revealed the potential of ML models in improving the accuracy of risk stratification of PTC patients Wang et al. (2022a).

MS-based multi-omics data can also be combined with ML for risk stratification in thyroid disease. Li et al. (2024) first reported a Preoperative Risk Assessment Classifier for PTC (PRAC-PTC) which constructed by ML models used clinical indicators, immune indices, genetic feature, and MS-based proteomics as multidimensional features. The results showed that six proteins (DPP7, PDLIM3, Col12A1, CTSL, TUBB2A, and ITGB5) were identified as the best discriminable proteins between low-risk and intermediate-risk/high-risk PTCs (Li et al., 2024). XGBoost showed the best performance among these ML models authors used. PRAC-PTC can increase the accuracy of the preoperative risk stratification and decrease unnecessary surgery or overtreatment.

Wang et al. (2024b) used different ML algorithms to explore the relationship between mixed-semi-volatile organic compounds (SVOCs) exposure and thyroid nodule. The data was collected by GC-MS/MS. RF and AdaBoost models were selected to screen out the features based on their contribution to the models. Weighted quantile sum (WQS) regression and Bayesian kernel machine regression (BKMR) were used to assess the mixed effects of the SVOCs exposure on thyroid nodule (Wang et al., 2024b). The results showed that high levels of exposure to SVOCs increase the risk of PTC and nodular goiters (NG), with Fluazifop-butyl and Fenpropathrin playing a major role (Wang et al., 2024b).

Wang et al. (2024c) proposed a ML-based objective method to individual to predict the risk of pediatric papillary thyroid carcinomas (PPTCs). They collected the clinical factors and MS-based proteomics data and nineteen proteins were selected by ML models to construct a protein-based personalized prognostic prediction model which can stratify PPTC patients into high- or low-recurrence risk groups and provide a suggestion for clinical decision-making and individualized treatment.

The combination of ML and MS-based multi-omics data in thyroid disease not only enhances disease risk assessment but also improves the approach to patient care. Through early intervention, patient stratification, and the implementation of targeted preventive measures, ML makes a significant contribution to improving patient health and building a more personalized and efficient clinical treatment system (Khalifa and Albadawy, 2024; Khalifa et al., 2024).

## 4 Summary

In the 21st century, the development of medical science has entered the era of big data, with ML algorithms, as a cornerstone of artificial intelligence, beginning to emerge in clinical disease research (Li et al., 2023b). From the clinical perspective, the application of ML in thyroid disease can contribute to the classification, diagnosis, treatment and prognosis. MS-based multi-omics analysis utilizing ML technology, offers significant prospects for early disease detection and prevention. However, there are currently limited examples of successful application of such technologies into clinical practice (Kelly et al., 2019). There are also many challenges must be addressed. In the biomarker field, the primary challenge is not in data analysis, but in the collection of comprehensive clinical data for each particular patient (Desaire et al., 2022; Arzyeh et al., 2020). The success of ML algorithms in the medical field largely depends on the quality, diversity, and completeness of the training data (Sarker, 2021). While the number of samples in some researches are not small, collecting more data will increase the diversity of patients. Models trained on broader and more diverse datasets will generalize better to new patients when deployed in real-world scenarios (Xi et al., 2022). Most researches mainly use publicly available datasets, and many datasets have the problem of class imbalance, with a very small number of samples in a certain class. Meanwhile, this phenomenon also leads to the results not being universal. When ML models are applied to such datasets, the models will overfit to the majority class, resulting in incorrect predictions for the minority class. The other limitation is that only a few types of thyroid diseases have been used to classification problems in existing studies, and most studies focus on binary classification, which has caused certain limitations in the application of ML to clinical applications of thyroid diseases. In disease research, this entails collecting detailed information about a patient’s medical history, genetic profile, treatment response, and long-term outcomes. Furthermore, patient privacy and data security must be carefully managed and addressed. As data volume increases, it is imperative to implement robust measures to protect patient privacy and uphold ethical standards (Abouelmehdi et al., 2018).

In summary, while ML holds substantial for clinical disease researches, it also encounters significant challenges to widespread adoption. Addressing these challenges will pave the way for realizing the full potential of ML in enhancing disease diagnosis, prognosis, and personalized treatment strategies.
